# Beyond stereotypes of adolescent risk taking: Placing the adolescent brain in developmental context^☆^

**DOI:** 10.1016/j.dcn.2017.07.007

**Published:** 2017-07-26

**Authors:** Daniel Romer, Valerie F. Reyna, Theodore D. Satterthwaite

**Affiliations:** a Annenberg Public Policy Center, University of Pennsylvania, United States; b Human Neuroscience Institute, Cornell University, United States; c Department of Psychiatry, Perelman School of Medicine, University of Pennsylvania, United States

**Keywords:** Brain development, Dopamine, Decision-making, Cognitive control, Experience

## Abstract

Recent neuroscience models of adolescent brain development attribute the morbidity and mortality of this period to structural and functional imbalances between more fully developed limbic regions that subserve reward and emotion as opposed to those that enable cognitive control. We challenge this interpretation of adolescent development by distinguishing risk-taking that peaks during adolescence (sensation seeking and impulsive action) from risk taking that declines monotonically from childhood to adulthood (impulsive choice and other decisions under known risk). Sensation seeking is primarily motivated by exploration of the environment under ambiguous risk contexts, while impulsive action, which is likely to be maladaptive, is more characteristic of a subset of youth with weak control over limbic motivation. Risk taking that declines monotonically from childhood to adulthood occurs primarily under conditions of known risks and reflects increases in executive function as well as aversion to risk based on increases in gist-based reasoning. We propose an alternative Life-span Wisdom Model that highlights the importance of experience gained through exploration during adolescence. We propose, therefore, that brain models that recognize the adaptive roles that cognition and experience play during adolescence provide a more complete and helpful picture of this period of development.

## Highlights

• Changes in the structure and function of the adolescent brain are placed in developmental context.
• Theories are challenged that posit adolescent imbalance between cognitive control versus sensation-seeking drives.
• Distinction is made between three forms of risky decision making, only one of which characterizes imbalance and only may apply to a subset of youth.
• An alternative Life-Span Wisdom Model highlights the adaptive characteristics of adolescent exploration and brain development.

## 1 Introduction

**Fig. 1:**
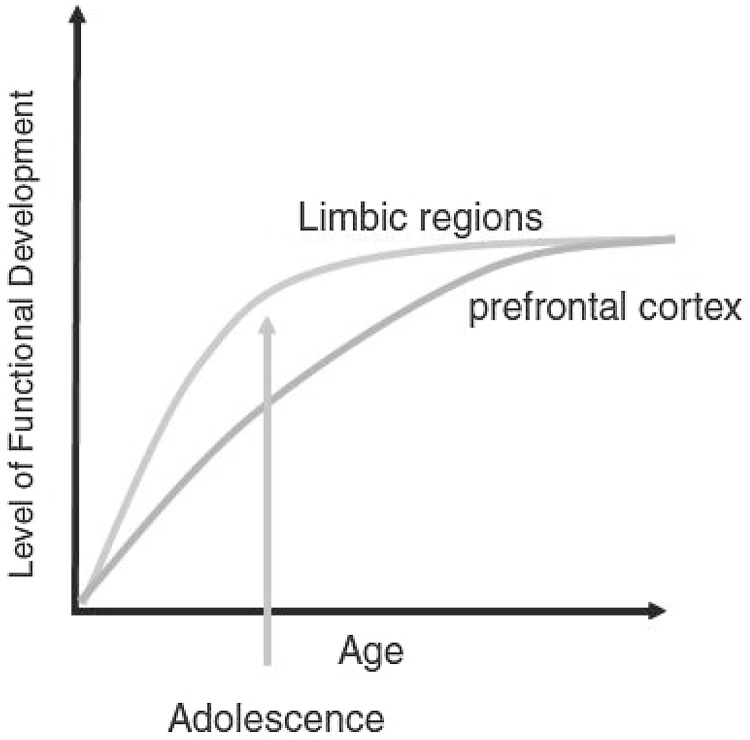
Casey et al. (2008) model of imbalance between prefrontal versus limbic control over behavior in adolescence.

Recent theorizing and research regarding the neurodevelopment of the adolescent brain has generated considerable attention in both the popular media and the scientific literature. The most striking generalization stemming from this work is that the adolescent brain does not fully mature until at least age 25, with the implication that adolescent decision-making and judgment is similarly limited up to this age (Casey et al., 2008, Giedd, 2004, Steinberg, 2008). This conclusion rests on research indicating that the myelination and pruning of the prefrontal cortex (PFC) continues into adulthood, well after ventral limbic regions that control motivation and reward have achieved these milestones. As a result, it is proposed that adolescents suffer from a structural as well as functional deficit in the ability of the PFC to exert top-down control over drives that are spurred by the limbic motivational system, leading to less than “rational” behavior during adolescence. The basic dynamics of these neurobiological imbalance models are illustrated in Fig. 1 (Casey et al., 2008), showing that limbic structures are activated in excess of prefrontal cognitive control regions during the adolescent period.

With permission from Institute of Medicine (2011, p. 38).

A key feature of such imbalance models is the suggestion that a developmental deficit in PFC cognitive control limits adaptive decision making by adolescents.^1^ However, when Giedd et al. (1999) first presented evidence of declining PFC gray matter volume in adolescents, they attributed the phenomenon to the role that experience plays in sculpting the brain during this developmental period. As they put it, the decline in PFC gray matter “may herald a critical stage of development when the environment or activities of the teenager may guide selective elimination during adolescence.” (p. 863). In other words, gray matter decline in the PFC could reflect pruning that results from the experience that adolescents gain during this period rather than a direct marker of increasing behavioral control. As Spear (2010) also noted, pruning may be “an example of developmental plasticity whereby the brain is ontogenetically sculpted on the basis of experience to accommodate environmental needs.” Needs could vary dramatically across environments and cultures (Mata et al., 2016), potentially resulting in very different patterns of pruning and brain organization during adolescent brain development (Choudhury, 2010). For example, evidence has accumulated to suggest that differences in socioeconomic status, which are correlated with cultural influences, are associated with differences in brain structure (Brito and Noble, 2014, Noble et al., 2015). In particular, Noble et al. (2015) demonstrated that lower socioeconomic status was associated with diminished cortical surface area and reduced hippocampal volume even when controlling for maternal education. Such hippocampal volume reductions have been reported by other studies as well (Hanson et al., 2011; Hueston et al., 2017). Others have observed differences in language-related regions (Piccolo et al., 2016) and modular brain organization (Krishnadas et al., 2013). Future research should unpack influences of education, culture, and income (with concomitant effects on nutrition, access to healthcare, and other factors that may plausibly affect development) on specific aspects of brain development.

Rather than emphasizing the important role of culture and experience in shaping the development of the brain, researchers have instead focused on excess levels of maladaptive risk behavior, such as injury, drug use, pregnancy, and other unhealthy outcomes, as support for imbalance (Dahl, 2004, Steinberg, 2008, Casey, 2015). However, the stereotype of the impulsive, emotional, and distraught adolescent rests much more on the *rise* in adverse outcomes during this age period than on their *overall prevalence* (Institute of Medicine, 2011, Rivers et al., 2008). For the vast majority of adolescents, this period of development passes without substance dependence, sexually transmitted infection, pregnancy, homicide, depression, suicide, or death due to car crashes (Institute of Medicine, 2011, Willoughby et al., 2013). Indeed, the risks of these outcomes are often comorbid with each other (Biglan and Cody, 2003, Kreuger et al., 2002), leaving the average adolescent without great risk of life-altering consequences.

We do not question the reality that the adolescent period entails risk. What we challenge is the interpretation of the brain and behavioral underpinnings of this risk. Research suggests that the brain is structured to enhance development by encouraging movement toward independence and self-sufficiency, a process that supports exploration and learning (Luna and Wright, 2015, Murty et al., 2016, Spear, 2013). Support for this view has been observed in both humans and other animals following the onset of puberty. Nevertheless, a focus on adverse outcomes leaves us with a biased picture that limits our ability to identify adaptive features of adolescent brain development within the context of the entire lifespan. Instead, we argue for a more nuanced interpretation of risk taking and its implications for healthy development. In particular, we outline the evidence regarding the role of sensation seeking, which although it peaks during adolescence does not reflect imbalance, as opposed to forms of impulsivity which either do not peak or only characterize a subset of youth. Our review of research regarding structural development indicates that the relation between brain structure and risk taking has failed to consider the implications of different forms of risk taking. Our analysis suggests that stereotypes of adolescents as particularly susceptible to unhealthy risk taking simplifies how adolescents think about risk and ignores the important role that experience plays in more adaptive forms of risk taking (Reyna et al., 2015a, Romer, 2010). In what follows, we consider what a broader perspective on adolescent brain development would suggest, how that helps to explain the way adolescents make decisions, and how these decisions can be improved.

### 1.1 The rise in sensation seeking

**Fig. 2:**
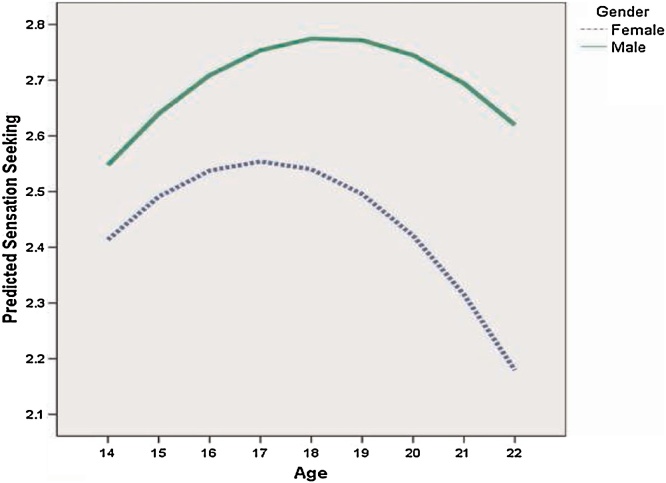
Trends in sensation seeking by gender in a national U. S. sample.

Consistent with stereotypes of young people, adolescents exhibit heightened attraction to novel and exciting experiences despite their evident risk (Chambers et al., 2003, Romer and Hennessy, 2007, Spear, 2010). This tendency, known as sensation seeking (Zuckerman, 2007), rises rapidly during adolescence. As seen in Fig. 2, a nationally representative U.S. survey of 1800 youth indicates that sensation seeking peaks around age 19 in males and 16 in females. A similar pattern has been observed across a wide range of countries (Duell et al., 2016). This rather striking pattern is regarded as a marker of rising dopaminergic activation during adolescence (Chambers et al., 2003, Wahlstrom et al., 2010) and may reflect activity in the midbrain dopamine pathway ascending from the ventral tegmental region (Ikemoto, 2007, Previc, 2009). This pathway traverses through the ventral striatum before branching into the orbital and ventromedial frontal cortex. These regions are heavily involved in recognition and anticipation of reward (Pagnoni et al., 2002, Schultz et al., 1997) and thus suggest a biological basis for increased attraction to novel and exciting experience during adolescence that declines as the brain transitions to adulthood (see Wahlstrom et al., 2010 for a review of evidence linking a peak in exploratory behavior during adolescence with changes in dopamine expression over the lifespan). A related personality cluster known as the behavioral activation system (BAS) is also believed to be related to dopamine function (Carver and White, 1994). One of the indicators of the BAS known as fun seeking is highly related to sensation seeking, while two other related indicators (reward responsiveness and drive) may be more associated with achievement motivations (Romer et al., 2016).

With permission from Romer (2010).

What is often neglected in discussion of imbalance is a rise in dopamine activity in dorsal and medial PFC (Meng et al., 1999, Weickert et al., 2007) fed by another pathway originating primarily in the substantia nigra that ascends through dorsal striatum into dorsal PFC and parietal cortex, regions that control movement and higher order decision making (Ikemoto, 2007, Previc, 2009). Dopamine neurons in this pathway appear to serve more global salience and cognitive processing functions than the ventral route (Bromberg-Martin et al., 2010, Roeper, 2013). This pathway enables the adolescent brain to exert greater attention and other executive functions that are important abilities for reasoning and complex decision-making (Cools et al., 2008, Cools and Robbins, 2004). In particular, dopamine is critical for the maintenance of activity in working memory (WM) (Arnsten et al., 2012, D’Esposito and Postle, 2015), a function centered in frontoparietal cortex that is critical for recruiting experience-based information during decision making (Fuster, 2009, Miller and Cohen, 2001, Shamosh et al., 2008). However, dopamine activation in the dorsal striatum has also been linked to various cognitive functions, including cognitive control and episodic memory (Bäckman et al., 2000; Bäckman et al., 2006; Volkow et al., 1998). Furthermore, as we describe below, both structural and functional dopamine activity in the striatum and PFC declines starting in the third decade of life with associated declines in these cognitive functions. Thus, the rise in dopaminergic activity that may underlie sensation seeking is also accompanied by increased dopaminergic activity in corticostriatal pathways that support the ability to exert control over rewarding experience and to learn from it (Murty et al., 2016; Whalstrom et al., 2010).

### 1.2 Brain development and adolescent self control

Emphasis on the reward-related functions of dopamine has reinforced a focus on impulsive behavior during adolescence. However, if the adolescent brain undergoes development in both ventral motivational and dorsal cognitive capacities, then the hypothesis of structural and functional imbalance as a normative developmental pattern needs reconsideration. Indeed, contrary to structural imbalance models of brain development, individual differences in sensation seeking (and associated risk taking) have been found to be *positively* correlated with WM and other indicators of executive function (Raine et al., 2002, Romer et al., 2011, Zuckerman, 2007). In one longitudinal study (Romer et al., 2011), individual differences in WM predicted subsequent levels of sensation seeking *even after controlling for age*, suggesting that sensation-based risk taking rises in concert with executive function. Indeed, executive function rises rapidly during adolescence (as does sensation seeking) and asymptotes well before age 25 (Gur et al., 2012, Luna et al., 2004, Williams et al., 1999). Thus, the rise in dopamine expression during adolescence may play a role in both sensation seeking and executive function.

Recent models of dopamine expression in mice and rats suggest that dopamine neurons become active in ventral and dorsal striatum prior to their emergence in medial PFC (mPFC) (Naniex et al., 2012, Reynolds et al., 2017). Indeed, dopamine pathways between orbitofrontal PFC and the striatum are in place prior to adolescence in humans (Fareri et al., 2015). The growth of dopaminergic connections between the striatum and mPFC is associated with improvements in cognitive functions related to value learning (Naniex et al., 2012, Reynolds et al., 2017). However, these gaps are eliminated by early adulthood, perhaps mirroring what happens in humans. As dopamine function in the mPFC grows during adolescence, there is also evidence that activation in the dorsal striatum is weaker than in the ventral region, a pattern that may have the adaptive function of enhancing exploration and action-outcome learning (Matthews et al., 2013). Nevertheless, many important cognitive functions that are subserved by the dorsal striatum and its connection with ventral PFC are in place prior to adolescence, and consistent with the development of cognitive control in humans, dopaminergic control over cognitive ability centered in the mPFC appears to be available by early adulthood.

**Fig. 3:**
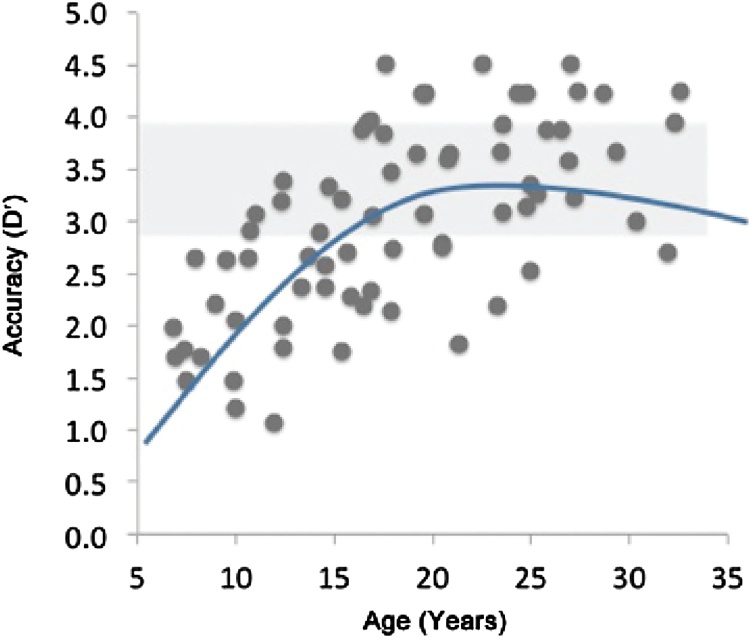
Data illustrating development of cognitive control during adolescence and early adulthood.

In view of the rise in both limbic and prefrontal dopamine expression during adolescence, the generalization that adolescents lack cognitive control relative to limbic activation may have been overstated, a conclusion also reached by Crone and Dahl (2012). Following their review of imaging studies of functional brain development, they found no pattern of brain activation that consistently distinguished adolescent from adult performance in cognitive control tasks: Some cognitive control tasks elicited higher activation in adolescents versus adults, whereas other tasks elicited lower activation. As seen in Fig. 3, by ages 16 and 17, the variability in executive control as assessed in a go-no task is already so large that many adolescents in that age range perform at a level that is equal to that of adults. Although early adolescents perform below the average level of adults in go/no-go and similar tasks, most late adolescents are either equal to or better than the average adult (Williams et al., 1999).

With permission from Casey and Caudle (2013).

**Fig. 4:**
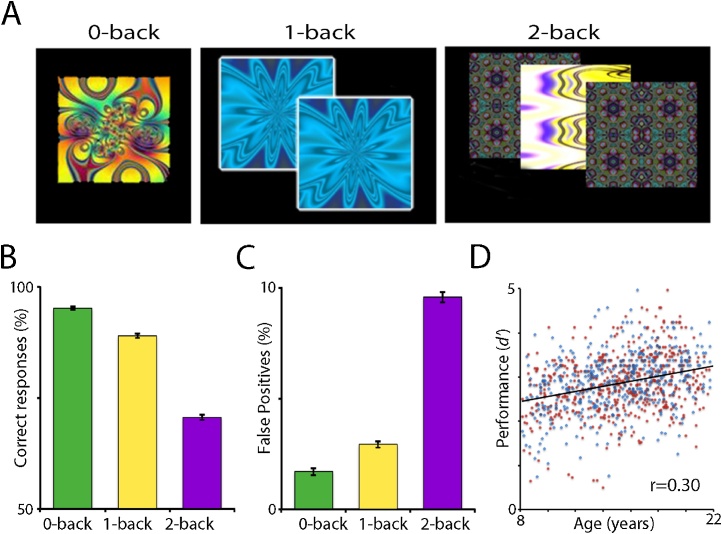
Data from Satterthwaite et al. (2013 with permission) illustrating the rise in WM ability from ages 8 to 22 that is overshadowed by individual differences. Panel A illustrates the stimuli used to assess different degrees of challenge to working memory. B and C show the increasing difficulty of the task as reflected in behavior. D shows the overall performance as measured with d’. Red points refer to females and blue to males.

Similarly, in one of the largest imaging studies of executive function in youth ages 8–22, Satterthwaite et al. (2013a) found that differences attributable to age were much smaller than individual differences in performance on an N-back WM task (see Fig. 4). Although WM improved with age, individual differences were large, with many late adolescents exhibiting better WM performance than the average young adult. Furthermore, brain scans demonstrated that WM performance was correlated with enhanced activation in PFC executive regions along with reduced activation of the default mode, which includes limbic cortex (Buckner et al., 2008; Raichle and Gusnard, 2005). Thus, while WM and executive function do improve with age in the aggregate, individual differences are large, such that many late adolescents are as capable as adults at recruiting performance-relevant activation of the executive system and deactivation of default mode regions.

### 1.3 Sensation seeking vs. impulsivity in adolescent development

A major contention of imbalance models is that adolescents are more subject to poor impulse control than either children or adults. For example, Casey (2015, page 299) notes that imbalance is “presumably not observed in childhood because there is a relative lack of maturity across and between regions within the (corticosubcortical) circuit, and in adults, there is a relative maturity of the underlying neurocircuitry.” However, in drawing this conclusion one must distinguish between sensation seeking, which does not appear to reflect imbalance, and impulsivity, which is a form of decision-making that is overly sensitive to immediate urges without adequate consideration of consequences. There are at least two forms of impulsivity that are relevant in regard to adolescent behavior (Winstanley et al., 2010). One called impulsive action reflects tendencies to act without thinking about consequences, also known as motor impulsivity (Patton et al., 1995, Romer et al., 2009). Acting without thinking is moderately positively related to sensation seeking, as well as the BAS and, also peaks during adolescence (Kasen et al., 2011, Shulman et al., 2015). A major difference between acting without thinking and sensation seeking is that, unlike sensation seeking, it is *inversely* related to WM ability (Khurana et al., 2012, Romer et al., 2011). This inverse relationship is understandable in that persons with this form of impulsivity lack the attentional control and capacity to consider alternatives to strong impulses.

Another form of impulsivity, known as impulsive choice (e.g., Mischel et al., 1989, Romer et al., 2010), reflects tendencies to choose smaller, immediate rewards over larger but delayed rewards (McClure et al., 2004, Metcalfe and Mischel, 1999). This preference for immediate reward is also inversely related to WM ability (Shamosh et al., 2008), again suggesting that weak ability to consider alternative courses of action predisposes to this form of impulsivity. Nevertheless, it is largely unrelated to sensation seeking (Cyders and Coskunpinar, 2011), which is not surprising given that it involves a choice between two rewards. Although it correlates with impulsive action, it does *not* exhibit a peak during adolescence. Rather it declines slowly from childhood onward, reflecting the increase in executive function during adolescence (Green et al., 1994, Romer et al., 2010, van den Bos et al., 2015). Thus, it is a simplification to assert that the adolescent period is marked by heightened impulsivity relative to children and adults considering that impulsive choice does not peak during this age period.

Although impulsive action and sensation seeking appear to conform to the stereotype of the impulsive adolescent, sensation seeking has different consequences from impulsive action. Research in both humans and other animals indicates that sensation seeking is positively correlated with PFC activation, while impulsivity displays the opposite tendency (Jupp and Dalley, 2014). Youth with high sensation seeking tendencies gravitate toward potentially risky activities, but in the absence of acting without thinking, they are less likely to experience adverse health consequences, such as addiction or problem gambling, than youth with impulsive tendencies (Khurana et al., 2017, Magid et al., 2007, Smith et al., 2007). These findings have remarkable parallels in the animal literature where it has been found that sensation-seeking lab rats are likely to try addictive drugs, but they are not likely to continue their use when it leads to adverse consequences (Belin et al., 2008, Winstanley et al., 2010). In contrast, rats that act impulsively are much more likely to develop addictive behavior that persists despite the maladaptive consequences. Lack of cognitive control, therefore, is more clearly characterized by impulsive action than sensation seeking.

Bjork and Pardini (2015) review the evidence regarding developmental changes in brain response to rewarding stimuli. Their review suggests that youth who exhibit harmful risk-taking tendencies exhibit brain responses consistent with weak cognitive control. However, this pattern is only representative of a subset of youth. Impulsive youth who lack self-control have been observed to display this characteristic at a young age and to continue to display poor control over behavior well into adulthood (Iacono et al., 2008, Moffitt et al., 2011). Indeed, such youth are disproportionately likely to experience the hazards that arise during adolescence and beyond, as examples, higher rates of injuries and illnesses due to automotive crashes, violence, and sexually transmitted infections (Moffitt et al., 2011, Sourander et al., 2006). Nevertheless, it is important for both theoretical and pragmatic reasons to distinguish risk taking that arises due to interest in exploring the environment from a developmental deficit in cognitive control during the adolescent period.

We have observed the beginnings of the distinction between exploratory and impulsive risk taking in a longitudinal cohort one of us is studying in Philadelphia (Romer et al., 2009). Youth ages 13–15 who began to use drugs with increasing frequency were much more likely to be highly impulsive than sensation seeking. Sensation seekers at this age try drugs, but they do not typically exhibit progression in regular use (Khurana et al., 2015a). A similar pattern was observed for early sexual initiation (Khurana et al., 2012) and unprotected sex (Khurana et al., 2015b). Although high sensation seekers may explore novel behavior that can lead to harmful outcomes if continued, they appear to learn from these experiences as they age, while youth with impulse control problems do not. These patterns suggest that the increase in sensation seeking that characterizes adolescence does not necessarily lead to maladaptive behavior unless it is accompanied by weak executive function, such as exhibited by acting without thinking or the desire for immediate reward.

As suggested by Reyna and Farley (2006), there appear to be two divergent routes to heightened adolescent risk taking: one that is associated with a *greater* reliance on executive resources (energized by a greater drive toward sensation seeking) and one that is associated with *reduced* executive capability (impulsivity) (see also Chassin et al., 1989, Reyna et al., 2015b. Despite the dominant narrative that adolescents are impulsive, Reyna and Farley’s (2006) overview of the literature suggests that much of adolescents’ risk taking is characterized by a surprising “rationality” in the conventional economic sense (Institute of Medicine, 2011). That is, risk taking across many real-world domains is found to be a function of trade-offs between perceived risks and benefits—as contrasted with impulsive or emotional risk taking. If anything, many adolescents can be described as “hyper-rational,” inasmuch as they rely on the risks and benefits of their behavior even more than adults do, which promotes risk taking when negative consequences are perceived to be unlikely (as is the case with many public health threats, such as contracting HIV).

Brain models that emphasize imbalanced development of limbic versus cognitive control regions suggest that adolescents are resistant to information about risks. Because the imbalance is “hard-wired,” there is little one can do other than to shield adolescents from their otherwise natural risk tendencies (Steinberg, 2008, Steinberg, 2014). However, trends in the use of both legal and illegal drugs, as assessed since 1975 by the Monitoring the Future (MTF) study (Johnston et al., 2015a), indicate that adolescents are responsive to the harm that drugs can pose. These harms are transmitted through various channels, including media campaigns (Elder et al., 2004), school-based education (Faggiano et al., 2008), and parental and peer influences (Bahr et al., 2005). Indeed, use of popular substances such as tobacco, alcohol, and marijuana have declined since the survey began. Furthermore, the correlation in the MTF study between annual rates of use of these drugs and perceptions of risk associated with those drugs was *r* *=* −0.83 for alcohol, *r* *=* −0.63 for marijuana, and *r* *=* −0.80 for cigarettes. These patterns are suggestive of an adolescent brain that is sensitive to adverse consequences despite interest in exploring novel experiences. Contrary to stereotypes about adolescents, Reyna and Farley’s (2006) overview of the literature showed that much adolescent risk taking was consistent with sensitivity to both perceived risks and benefits, which is a rational rather than impulsive process according to traditional views of rationality.

### 1.4 The importance of type of risk

Research concerning the imbalanced adolescent brain has taken a rather broad brush approach to the assessment of risk taking. In this section, we review what is known about developmental trends in risk taking as assessed in laboratory tasks and how different forms of risk taking are related to cognitive control. As previously noted, despite stereotypes of adolescents as more impulsive than either children or adults, there is considerable evidence that some risk-taking preferences (such as impulsive choice) do not peak during adolescence but instead follow a monotonic decline from childhood to adulthood. A developmental decline in risk taking is common in tasks in which the gains and losses attributable to different choices are explicitly defined or able to be learned quickly (Defoe et al., 2015). This kind of task, known as decision under risk, is different from ones in which the outcomes and associated probabilities are ambiguous or unknown, commonly known as decisions under ambiguity (Brand et al., 2006, Volz and Gigerenzer, 2012).

Assessments of impulsive choice fall under the rubric of decision under risk in that these paradigms explicitly provide information regarding the magnitude of reward and the likelihood of its occurrence as denominated by either delay or probability. Other tests of decision under risk provide choices between two or more alternative options that differ in reward and probability of outcome. A common task is one in which a certain positive option is contrasted with a riskier option even though the expected value of the risky option is equivalent to the certain option (e.g., win $2 for sure vs. equal chance to win nothing or $4) (see Levin and Hart, 2003). These tests also demonstrate a monotonic decline in risk taking in which children are *more* risk seeking than adolescents who are more risk seeking than adults. When different age groups are compared on other types of choice tasks in which a certain option is not available, the same decline is evident once IQ is held constant (Defoe et al., 2015). This control is important because it is difficult to arrange choice tasks that are understandable for children (e.g., under age 10; see also Reyna and Ellis, 1994).

Like Levin and Hart (2003) and Reyna and Ellis (1994), Paulsen et al., 2011, Paulsen et al., 2012 designed a task that was easily comprehended from childhood to young adulthood and found clear evidence for a decline in risk seeking whether a certain option was available or not. One explanation for this clear deviation from imbalance models as well as stereotypes of adolescent impulsivity is that adolescents are more risk seeking under ambiguity than children or adults (Paulsen et al., 2012). That is, given the potential for a reward but lack of information about its likelihood, adolescents will be more inclined to *explore* the risky option than either children or adults. As a result, they may actually exhibit a more rational response than adults who are notoriously risk averse when certain rewards are at risk.

In a demonstration of adolescent exploration, Tymula et al. (2012) showed that compared to adults, adolescents are more likely than adults to take risks that are ambiguous. As a result, their behavior was more “rational” in the economic sense of evaluating options based on expected value than adults. In their study, adults were so averse to unknown risks that they preferred expected values that were far smaller than adolescents were willing to tolerate. As they conjectured, “such a tolerance may make sense, because it would allow young organisms to take better advantage of learning opportunities.” Adolescents’ greater tolerance for ambiguity may also reflect their overly optimistic evaluation of the rewards of novel behavior (Romer, 2010, Romer and Hennessy, 2007). Exploration of novel environments has survival value and has been linked to activity in both PFC and subcortical regions (Daw et al., 2006), again suggesting that adolescents may not be structurally handicapped with respect to specific information-processing abilities that facilitate learning.

**Fig. 5:**
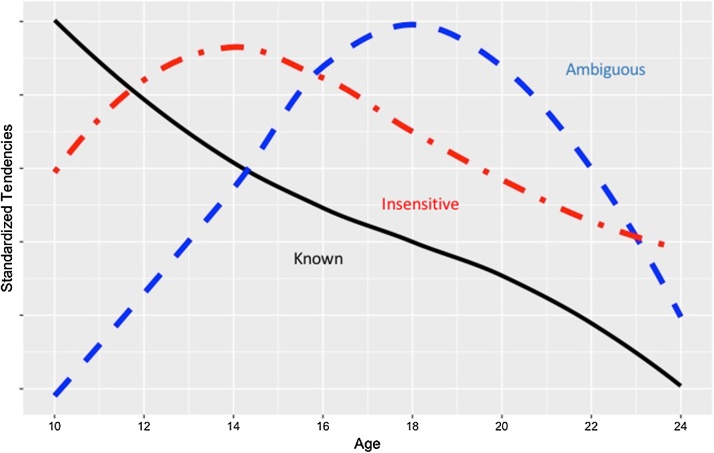
Differences in three types of risk taking tendencies across age. Trends for Known and Ambiguous risks apply to all adolescents while the trend for Insensitive risk taking applies to youth with high levels of acting without thinking that precede adolescence and remain elevated into adulthood.

The evidence we have reviewed suggests that in characterizing adolescent risk taking, it is critical to distinguish between different types of risk behavior, each of which has unique motivational and cognitive underpinnings. We describe these different patterns in Fig. 5. Impulsive action is characterized by *insensitivity* to risk, a form of risk taking that peaks during early adolescence. However, it is only characteristic of a subgroup of youth with weak executive function and self-control, conditions that are present prior to adolescence (Bjork and Pardini, 2015, Kreuger et al., 2002, Moffitt et al., 2011). This form of risk taking is most clearly associated with the behavior that imbalance models seek to explain. In the absence of intervention, this form of imbalance can persist into adulthood. Impulsive choice as well as other forms of decision making under *known* risk do not peak during adolescence. Indeed, adolescents are more inclined to avoid risks than children under delay of reward or other forms of decision making under known risk. Finally, choice under *ambiguity* is sensitive to sensation seeking tendencies that encourage exploration, such as use of drugs (Romer and Hennessy, 2007). Although it may peak during adolescence, exploration and tolerance of ambiguity is not devoid of cognitive control and may actually be more adaptive in many circumstances than the extreme ambiguity avoidance exhibited by adults.

### 1.5 Do adolescent risk taking tendencies match predictions of imbalance?

If developmental imbalance between cognitive control and limbic activation were responsible for peaks in adolescent risk taking, one would expect those peaks to occur in mid-adolescence when imbalance is at its height (Willoughby et al., 2013). However, to the degree adolescents suffer injury, the period of highest risk occurs rather late in the transition to adulthood when inexperience is still high despite the nearly complete maturation of brain networks that are thought to enable cognitive control. For example, use of either cigarettes or marijuana peaks around age 20 in the U.S. (Romer, 2010); while binge drinking remains high throughout the third decade (Johnston et al., 2015b). Deaths due to overdoses of alcohol peak much later, around age 50 (Kanny et al., 2015), although younger drinkers may be more likely to overdose and survive. The proportion of driving fatalities attributable to alcohol peaks between ages 21 to 34 and continues at high rates until age 54 (US Census Bureau, 2012). Sexually transmitted infections such as gonorrhea and chlamydia peak between ages 20 to 24 (Centers for Disease Control and Prevention, 2014).

Conclusions about age trends in risk-taking must take risk opportunity and other co-occurring factors into account, as Shulman et al. (2016) note. In particular, research has shown that adult supervision of adolescents declines as they age, especially in males, thereby providing increasing opportunity to take risks (Gerard et al., 2008). However, with sensation seeking on the decline and cognitive control at its peak during early adulthood, any increases in unhealthy risk taking during this period would seem less attributable to imbalance than to stable individual differences in impulsivity that emerge prior to adolescence and remain evident into adulthood (Iacono et al., 2008, Moffitt et al., 2011). As adolescents enter young adulthood, they are presented with even greater risk-taking opportunities that will challenge those with weak cognitive control.

In summary, the appeal of the imbalance model rests in part on the popular stereotype of the adolescent as impulsive and lacking in cognitive control. Closer examination of this stereotype reveals that only one form of impulsivity (acting without thinking) peaks during adolescence and that this form of impulsivity varies significantly across individuals. The other major form of impulsivity, impulsive choice, declines from childhood to adulthood and thus is not likely to be explained by peaks in adolescent imbalance. In addition, other decisions under conditions of known risk also decline from childhood to adulthood. Finally, although sensation seeking does peak during adolescence, it is not characterized by the absence of cognitive control.

### 1.6 Evidence for structural imbalance

If imbalance does not characterize all types of risk taking behavior, what is the evidence regarding structural imbalance in brain development? Studies of brain structure and risk taking tend to produce confusing results, which is not surprising given that risk taking itself is a complex behavior. Some forms of risk taking can be positively related to executive function (e.g., decision under ambiguity) and others inversely related (e.g., impulsive action).

In normal development, gray matter loss in PFC is thought to be a marker of maturation, perhaps reflecting fine-tuning of brain structure (Spear, 2010). However, research examining structural brain development in relation to executive control has found that *less* prefrontal gray matter is associated with ADHD and forms of impulsivity that emerge early in development (Shaw et al., 2011; van Ewijk et al., 2012). Such persons also exhibit a lower rate of gray matter reduction as they age. With thinner cortical gray matter at the outset of adolescence, there may be less to prune. Thus, simple indices of gray matter reduction are unlikely to be a pure marker of enhanced cognitive control. Indeed, the development of brain structure varies with IQ in a complex interaction with age. Shaw et al. (2006) demonstrated that higher IQ is associated with *thinner* cortex in childhood, while in adolescents this relationship is reversed and *thicker* cortex is associated with higher IQ. A more recent longitudinal study of 504 participants corroborated this interaction with age, but suggested that the transition point may occur in early adulthood (age 21) rather than adolescence (Schnack et al., 2015).

Recent research suggests that patterns of gray matter change are location dependent and underlines the importance of white matter expansion occurring as a result of myelination. Vandekar et al. (2015) recently showed that gray matter reduction was maximal in sulci where white matter organization occurred. Multivariate analyses also revealed a second pattern, whereby gyral cortex thickened in early adolescence, a process that appears to asymptote by age 13. Thus, while this finding awaits replication in longitudinal studies, human neuroimaging research indicates that cortical *thinning* may be the result of both myelination and pruning, while cortical *thickening* characterizes a secondary maturation pattern that occurs during adolescence in more localized parts of gyral cortex. These more complex patterns of gray matter change further suggest that indices based on overall gray matter change are likely to obscure more complex organizational changes in brain structure as adolescents age. Indeed, it appears that the dominant pattern of brain development from childhood to adulthood is monotonic decline in gray matter associated with increases in myelination (Lebel et al., 2012). However, Berns et al. (2009) found that controlling for age, white matter maturation was *positively* related to a wide range of prior real-world risk behavior in adolescents, some of which may well be associated with exploratory drives. This pattern was recently replicated in an experimental context by Kwon et al. (2015). Nevertheless, other research finds that white matter integrity in some brain regions is weaker in children with ADHD, suggesting that some white matter deficits play a role in youth with this form of impulsivity (van Ewijk et al., 2012). In sum, there does not appear to be a simple relation between myelination and risky behavior. Developmental differences in myelination can be associated with *greater* rather than less risky behavior during adolescence, especially when the risks are ambiguous. On the other hand, conditions such as ADHD which are likely to reflect impulse control problems may be characterized by less white matter development.

Analyses of gray matter maturation in limbic regions also fail to conform to expectations of structural imbalance. Rather than reflecting early maturation in limbic structures, gray matter change continues well into adolescence (Dennison et al., 2013, Raznahan et al., 2014). A direct test of the structural imbalance model conducted by Mills et al. (2014) examined differential brain maturation in a longitudinal study of volume changes in the PFC versus the amygdala and the nucleus accumbens. Using three scans across childhood, adolescence, and young adulthood, these researchers found that the amygdala exhibited *increased* volume up to about age 16, when growth in this structure began to asymptote. The accumbens exhibited declining volume as adolescents aged. Using these limbic regions as indicators of imbalance in relation to maturation of the PFC, the researchers correlated individual differences in structural imbalance with reports of real-world risk taking. Consistent with the possibility that the risk taking recalled by those participants was a mixture of exploratory and impulsive behavior, there was no correlation between the imbalance observed in brain structure and reports of risk behavior during adolescence. Notwithstanding the study’s sample size (n = 33), the authors “failed to find a relationship between the presence of a mismatch in brain maturation and risk-taking and sensation-seeking behaviors during adolescence.” (p. 147).

The imbalance model advanced by Galván et al. (2006) suggested that ventromedial PFC (vmPFC) matures more slowly than the ventral striatum and that greater activation in the striatum relative to vmPFC could be the source of greater risk taking in adolescents. This model does not seem to follow from the original observation that brain maturation during adolescence proceeds from ventral to dorsal regions. Indeed, a recent study examining resting state connectivity between the striatum and ventral- and medial-PFC found that these regions exhibited early and rather stable connectivity from childhood to adulthood (Fareri et al., 2015).

It is important to note that Galván et al. (2006) based their conclusions on a reward learning paradigm in which adolescents have been shown to exhibit greater ventral striatal response to reward prediction errors than adults (see also Section 2.2 of this issue later in regard to Cohen et al., 2010). Thus, this heightened striatal response may not be a particularly sensitive indicator of maladaptive risk taking. Furthermore, as participants gained experience in the task, adolescents also showed an anticipatory vmPFC response suggesting that this region “predicted” the outcome of the reward cue, an ability that is associated with healthy vmPFC function (Rolls, 2014). Thus, if anything, this study showed greater functional synchrony between these regions in adolescents than in either children or adults, a finding potentially indicative of greater sensitivity to reward learning. A follow-up study by Galván et al. (2007) found that heightened ventral striatal activation in receipt of reward was a predictor of the likelihood of engaging in hypothetical real-world risk-taking; however, this was an individual difference, characteristic of both adults and adolescents.

Christakou et al. (2011) found that activation in vmPFC and connectivity with ventral striatum was related to age-dependent decline in impulsive choice. Consistent with the cognitive control predictions of imbalance models, but not the reward sensitivity predictions, this form of risk taking did not peak during adolescence. Thus, this study did not directly address the conditions underlying adolescent-specific imbalance.

In total, the findings suggest that white matter development and associated declines in gray matter are not clearly related to reduced risky behavior. Furthermore, connectivity between the striatum and vmPFC is established early in development such that adolescents need not be handicapped by inadequate linkage between these regions. Indeed, the evidence appears to be more consistent with the important role of the vmPFC in reward-based learning during adolescence, and the close connectivity between this region and the ventral striatum (Haber and Knutson, 2010, Rolls, 2014).

### 1.7 Other models of risky decision making

Other models of risky decision making also focus on the relative strength of cognitive control and reward sensitivity processes (Casey, 2015). However, these models do not require a structural deficit in the ability to exercise self-control. For example, McClure et al. (2004) find that within the same individuals, making less impulsive choices is associated with greater activity in PFC cognitive control regions, while during the same scanning session making impulsive choices is associated with ventral striatal activation. Nevertheless, in a recent developmentally sensitive study across ages 8–25, van den Bos et al. (2015) found that functional connectivity between medial striatum and cognitive control regions (dorsolateral and ventrolateral PFC) mediated declines in impulsive choice across age. However, as has been observed in other research (Green et al., 1994, Romer et al., 2010), discount rates declined rapidly from childhood to early adolescence and showed very little decline from that point onward. Thus, apart from individual differences, impatience may not be particularly relevant for understanding adolescent peaks in maladaptive adolescent risk taking. In addition, connectivity change was observed with the medial rather than ventral striatum, suggesting greater involvement with cognitive control than motivational functions of the striatum (Cools et al., 2008), a result consistent with the finding that sensation seeking and discounting are largely uncorrelated (Cyders and Coskunpinar, 2011, Romer et al., 2010).

The model of hot versus cold cognition proposed by Metcalfe and Mischel (1999) proposes that reducing the appeal of immediate (hot) rewards can be accomplished by flexible allocation of attention (e.g., thinking about something other than the reward). This model also focuses on the ability to delay gratification, a form of impulsive choice that does not peak during adolescence. Although the ability to allocate attention may increase with development, it is not a skill that is particularly impaired in adolescence relative to earlier ages, and variation in tendencies to delay gratification may well be driven by individual differences in life experience (McGuire and Kable, 2013).

The Driven Dual Systems model proposed by Luna and Wright (2015) also focuses on imbalance between cognitive control and dopamine driven reward motivation. Unlike the Casey model in Fig. 1, their model recognizes that cognitive control achieves adult levels by mid-adolescence. However, they suggest that the rise in dopamine activation during adolescence exceeds the levels experienced by adults, thereby predisposing toward immediate rewards in excess of adult levels. Nevertheless, Luna and Wright suggest that the sensation seeking that results from this imbalance has adaptive characteristics, such as the need to explore the environment. They also note that this imbalance “may make some adolescents vulnerable to risk-taking behavior’ (p. 107). Luna and Wright use the term risk-taking to characterize maladaptive behavior by definition; but as we have noted, exploration is a form of risk-taking that need not be maladaptive. Thus, their model is consistent with our suggestion that the rise in maladaptive risk taking only characterizes some adolescents and thus accords with the analysis presented here.

Another model that has garnered significant attention in regard to adolescent brain development is the Triadic Model of Ernst and colleagues (Ernst, 2014, Ernst and Fudge, 2009). This model is described by Ernst (2014) as a ‘heuristic tool’ for organizing neuroscience research on motivated behavior. The model not only considers imbalance between cognitive control and reward processing regions but also includes potential imbalance with avoidance processes centered in the amygdala and related regions. Ernst proposes that the three regions act to achieve an equilibrium that “varies across individuals.”

The triadic model rightly expands the brain regions that must be considered in understanding developmental changes during adolescence. However, although the amygdala has input to the ventral striatum, it is sensitive to both rewarding and aversive events. Rather than serving to balance the ventral striatum, it may actually alert the ventral striatum to salient events that require action (Rolls, 2014). In their reviews of literature regarding reward processing, Richards et al. (2013) also note the wide differences that obtain depending on the laboratory task and the incentives provided to research participants. In some paradigms, adolescents exhibit control equal to adults, while in others they do not. However, even when adolescents appear to engage in less cognitive control than adults, this deficit can be overcome by increasing incentives for performance (Richards et al., 2013). In sum, the model may apply more to individual differences due either to experience or tendencies that exist prior to adolescence.

Casey (2015) also suggests that models of adolescent risk taking include interconnections between more than just the striatum and PFC. She highlights findings suggesting that compared to children and young adults, adolescents exhibit stronger emotional responses to laboratory stimuli. For example, adolescents commit more errors in a go/no-go task when the no-go cue is a smiling face compared to a neutral face. What is less clear is how these responses relate to real world risk taking. It may be that such responses are related to exploratory behavior, which is less likely to lead to harmful consequences than high levels of impulsive behavior. Other examples of emotional responses to emotional stimuli suggested that in some paradigms (but not others), adolescents react more strongly to aversive stimuli, such as fearful faces. But here again, it is not clear that these responses would lead to heightened or harmful risk taking, and in some cases, heightened adolescent response only characterized some adolescents, with others showing emotional responses comparable to children and adults (Hare et al., 2008). At this point, without the necessary clarifying information regarding the type of risk taking that is being examined, it is difficult to draw conclusions about such evidence.

In summary, our review of the evidence regarding structural differences in brain development suggest that the adolescent brain undergoes rapid change during this age period, but connections to maladaptive risk behavior depend on both individual differences and the type of risk taking. Evidence linking brain structure and function to risky behavior tends to be inconclusive regarding imbalance, and this is not surprising given the many ways that risk taking can manifest. Furthermore, cognitive control reaches maturity by early adulthood when sensation seeking is in decline but the adverse effects of risk taking begin to peak. Thus, the developmental imbalance that is suggested to be at the root of such adolescent risk taking is unlikely to explain this rather late appearance of developmental risk. We propose instead that for the majority of adolescents, maladaptive risk taking declines from childhood on. For those with heightened impulsivity, risks can continue to grow as opportunities for such behavior increase; however, this pattern is concentrated in a subset of youth who exhibit impulsive behavior prior to adolescence.

## 2 Cognitive control vs. experience-based cognitive development over the lifespan

Imbalance models suggest that cognitive control develops linearly during adolescence while sensation seeking peaks. Furthermore, Shulman et al. (2016) claim that cognitive control continues to grow well into young adulthood and that this helps to explain the continued rise in risk-taking during this period. Here we note that this presumed linear increase in cognitive control conflates two separate cognitive processes, one based on structural maturation of the cognitive control system and the other dependent on increasing connectivity between the PFC and parietal, occipital, and temporal cortices that build over time with experience (Fuster, 2009, Fuster, 2013). When these are separated, it becomes clear that cognitive control also peaks by late adolescence and early adulthood while experience-based development continues in a monotonic fashion well into the aging process.

The distinction between cognitive control and experience-based cognitive development is consistent with recent research that has moved beyond simple models of gray matter change to more nuanced analyses of brain networks (Ernst et al., 2015; Pfeifer and Allen, 2012). An important study by Dosenbach et al. (2010) examined the development of functional brain networks from ages 7 to 30 using resting-state fMRI. Using a machine-learning approach, they showed that measures of functional connectivity could provide an index of brain network maturation that correlates with age. The most important features of this model are enhanced connectivity *within* large-scale functional brain networks, such as the executive control and default mode networks, but reduced connectivity *between* such networks during the adolescent age period (Baum et al., 2017, Stevens, 2016). Interestingly, analyses indicate an asymptote in functional network development by age 22, before presumed maturation of pruning and white matter growth has run its course. However, the dataset was somewhat sparse in the late adolescent age range, leaving open the possibility that the asymptote occurred even earlier (e.g., see Vaso et al., 2017). In addition, similar to the pattern of WM development observed by Satterthwaite et al., 2013a, Satterthwaite et al., 2013b, the range of maturation of brain networks during the resting state varied widely across individuals. These patterns have been subsequently replicated in independent datasets controlling for confounds due to head movement (Fair et al., 2013, Satterthwaite et al., 2013b).

Rubia (2013) and Luna et al. (2010) summarize the changes in brain activation that occur in cortex from childhood to later adulthood. Their summaries indicate increasing connectivity within cognitive control networks as children age, which may contribute to greater cognitive control during adolescence. This conclusion is consistent with recent studies indicating that brain networks involved in cognitive control versus default mode become more segregated during adolescence (Baum et al., 2017, Dosenbach et al., 2010, Fair et al., 2008), but conversely become less segregated during later adulthood, *thereby displaying an inverted-U shaped pattern of interconnectivity across the lifespan* (Betzel et al., 2014, Chan et al., 2014). Furthermore, Chan et al. (2014) found that reduced network segregation at any adult age was associated with an important marker of age-related cognitive decline, namely weaker verbatim memory. As summarized by Betzel et al. (2014), on the one hand, functional connectivity (FC) over the lifespan *within* resting state networks (RSNs) “decreased with age, affecting higher-order control and attention networks. On the other hand, FC tended to increase *between* RSNs, especially among components of the dorsal attentional network, the saliency/ventral attention networks and visual and attention networks and the somatomotor network.” (p. 352).

These changes are consistent with a brain that grows in cognitive ability during adolescence but that increasingly relies on between-network connections as adulthood progresses into aging. For most adults, the ability to exert cognitive control or behavioral inhibition eventually declines as indexed by tasks that challenge response speed and attentional skills (e.g., stop-signal and WM) (Lindenberger, 2014). However, older adults have greater ability to draw from experience, which is consistent with growing connectivity between networks.

### 2.1 The importance of experience

Despite the stereotype of adolescents as impulsive risk takers, it is important to consider the role of exploration and learning that occurs during this period of development. Fuster, 2009, Fuster, 2013 proposes a model of brain development across the neocortex involving what he calls *cognits* or networks of neuronal connections between the PFC and other cortical regions that build over time. Cognits provide a bridge between “executive memory” in the PFC and sensory and “perceptual memory” in other regions. These memories enable a form of what Goldberg (2006) calls “executive intelligence” built from experience in encountering novel problems. These networks are assumed to develop in a hierarchical manner, such that individual experiences reside at the lowest level of the network. As experience accumulates, more abstract levels of memory are formed that enable clearer decision rules for action across similar domains. These more abstract memories provide experienced actors with shortcuts to decision-making that require less cognitive effort than less experienced adolescents might have to exert.

Fuster’s theory of cognits is also broadly consistent with fuzzy-trace theory advanced by Reyna and colleagues, who highlight the importance in decision-making of a distributed system of gist in the brain, as opposed to localized verbatim, memory representations (Reyna et al., 2015b; see Reyna and Huettel, 2014, for differences in neural substrates). Fuzzy-trace theory emphasizes the accumulation of experience that leads to more adult-like decision-making and *gist-based intuition* (Reyna and Brainerd, 2011, Wilhelms et al., 2015). As people gain experience in a decision domain, they begin to *understand* patterns in the outcomes that accrue, a process that enables them to rely on more abstract gist principles regarding those decisions and less on the literal rewards and costs of a particular decision. This experience encoded in durable gist memories would be expected to facilitate decision-making (Fuster, 2009, Goldberg, 2006, Reyna and Lloyd, 2006, Reyna and Mills, 2014). Although late adolescents and young adults have greater cognitive control than the average older adult, they may not have developed the insight from experience, or what is conventionally called wisdom, that is important for functioning in the world (Reyna et al., 2011). Such experience would convert many ambiguous risk situations to ones with known risks that elicit less risk taking with age.

Research on cognition has shown that people mentally represent information about decision options in two ways: verbatim representations of details, which are precise enough to support analytical thinking, and gist representations, which are less detailed (i.e., fuzzy) and support impressionistic, parallel, and typically unconscious thinking (similar to characterizations of intuition; Reyna, 2012). The preference to rely on gist grows with experience, and, for risk and probability, the simplest gist is categorical, for example, the categorical distinction between some risk or no risk (e.g., Reyna et al., 2014, Reyna and Ellis, 1994). As adolescents age, it would be expected that they would also increasingly rely on gist-based reasoning when confronted with potentially maladaptive risk taking. The growth of reliance on more abstract gist memories from childhood to adulthood, as predicted by fuzzy-trace theory, has been replicated in 53 out of 55 studies on gist-based “false” memories (Reyna, 2011).

Consistent with a monotonic decline in risk taking with unambiguous risks, there is evidence that adolescents with better executive function perform better on such tasks (Brand et al., 2006, Khurana et al., 2015a, Shamosh et al., 2008). This evidence suggests that the decline that occurs with development can be attributed in part to increasing ability to store and compare outcomes of risky decisions. Such ability would also lead to better integration of experience when confronting risky situations, including reliance on gist-based memories. As a result, preference for maladaptive risk taking in specific domains would be expected to decline as experience accumulates and to do so more rapidly for youth with better executive function.

The meta-analysis by Defoe et al. (2014) (see also Tymula et al., 2012) contrasted the predictions of fuzzy-trace theory versus imbalance theories in laboratory tests of risk taking. The finding that risk taking declines with age, especially when a certain option is available, is not anticipated by imbalance theories. The presence of a certain versus risky option provides a critical test of contrasting theoretical predictions (e.g., see Kühberger and Tanner, 2010). Fuzzy-trace theory predicts that a gist representation favors the selection of the certain option for gains, a preference that grows with experience. Experiments on the development of risk taking confirmed that, in addition to motivational and cognitive control factors, risk preference is a function of competing verbatim versus gist mental representations of decision options. From the perspective of gist-based intuition, risking HIV infection by having unprotected sex is a bad idea even if the risks are low and the benefits are high (see Reyna, 2008). These theoretical ideas explain the otherwise puzzling (but predicted and replicated) result that experience, both from childhood to adulthood and from novice to expert in a specific domain of decision making, is associated with greater reliance on gist-based intuition rather than verbatim reasoning (e.g., Reyna et al., 2011, Reyna and Lloyd, 2006).

The greater verbatim information-processing efficiency of adolescents (relative to children and aging adults) would appear to be a benefit that compensates for their lack of experience. Adults progressively lose the ability to exert cognitive control over their attention and WM capacities (Lindenberger, 2014), leading to what Goldberg (2006) has termed “The Wisdom Paradox.” With aging, the neocortex continues to lose gray matter in PFC with associated reductions in the ability to remember verbatim details of past experience and to hold information in WM (Chan et al., 2014; Dennis et al., 2013). Adults experience a domain-general decline in verbatim cognitive skills starting in the third decade of life (Tucker-Drob, 2011, Lachman et al., 2014), although gist memory is conserved (e.g., Brainerd et al., 2009; Reyna, 2012). During the same period of verbatim decline, the brain is estimated to lose about 7% of its striatal dopamine transporters per decade (Volkow et al., 1996), with even larger declines in the PFC (Eppinger et al., 2011). These declines, which begin in the third decade of life, are associated with reductions in various cognitive and motor functions, including episodic and working memory, inhibitory control, and switching (Bäckman et al., 2010; Li et al., 2010; Volkow et al., 1998). Yet, consistent with conservation of gist-based intuition, older adults’ risky decision-making remains largely indistinguishable from that of younger adults when verbatim memory is not required (Mata et al., 2011; Samanez-Larkin and Knutson, 2014). Although adults are able to make good, and perhaps even better decisions than adolescents, they rely on their accumulated experience to counterbalance the declines in executive function that they once possessed in late adolescence and early adulthood.

From the perspective presented here, experience making risky decisions during adolescence, as executive functions develop, fosters increased development of gist-based reasoning. This experience is especially critical because it allows adults to avoid unhealthy risks using cognitive capacities (i.e., gist memory) that are preserved over a lifetime and that are robust in stressful or emotional situations (e.g., Reyna and Brainerd, 2011; see Reyna, 2011, for estimates of verbatim and gist memory, as well as cognitive control, across the lifespan). The growth in this ability reflects increasing wisdom, defined as the accumulation of gist-based insight and expert knowledge about the conduct and management of life challenges (Baltes and Smith, 2008, Baltes and Staudinger, 2000, Sternberg, 2001).

From a neurodevelopmental perspective, wisdom most likely involves the maturation (including pruning) and interconnection of several brain regions that enable the individual to harness experience in an adaptive fashion (Meeks and Jeste, 2009, Reyna and Huettel, 2014). These include the executive control and limbic systems. The default mode network including medial PFC plays an important role by facilitating self-referential processing, empathy, theory of mind, and future projection (Buckner et al., 2008, Meeks and Jeste, 2009). As noted, this system exhibits increasing intra-connectivity during adolescence (Fair et al., 2008, Sherman et al., 2014, Supekar et al., 2010). Nevertheless, it is the integrated functionality between systems across development that distinguishes wisdom from a simple top-down impulse control system (Reyna et al., 2015b).

**Fig. 6:**
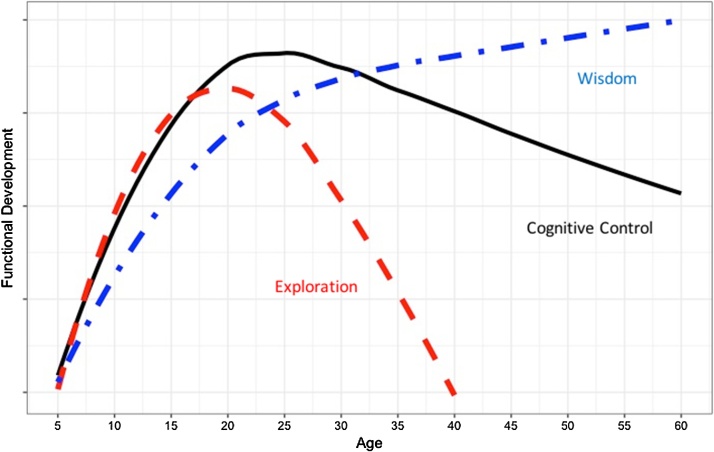
Hypothesized trajectories of the Life Span Wisdom Model of cognitive control, exploration, and experience. Y axis scale is arbitrary.

We summarize the changes that occur relevant to adaptive decision making over the lifespan in Fig. 6. This model differs from imbalance models in several respects but most importantly by including a third trajectory representing the accumulation of experience and hence wisdom. Executive function displays an inverted U-shape function that peaks in late adolescence and early adulthood (Lachman et al., 2014, Lindenberger, 2014, Williams et al., 1999). At the same time as executive function is improving during adolescence, the rise in sensation seeking and related dopamine expression drives exploration of the environment which peaks earlier than executive function but subsides during later years (Mata et al., 2016). However, as we describe below, as a result of these two processes, the brain builds networks of experience that foster greater ability to make adaptive decisions in later adulthood despite the decline in executive function (Richards and Hatch, 2011, Webster et al., 2013). Thus, the rise in exploration that characterizes the adolescent brain serves an adaptive purpose of building robust representations of experience.

The model also recognizes that the late adolescent and young adult brain is still a work in progress during the period when exploration and wisdom are growing despite having reached the peak in cognitive control. Thus, late adolescents and young adults will still be exploring their world during this period and taking risks many of which can be adaptive. For those with especially weak cognitive control however, this period may produce particularly unhealthy consequences, such as addiction and unintentional injuries, many of which were foreshadowed by earlier impulsive behavior.

### 2.2 The adaptive adolescent brain

Although heightened sensation seeking makes novel and potentially risky behavior more common during adolescence, this risk taking may be motivated by a “rational calculus” (Reyna and Farley, 2006) that may be adaptive for learning that underlies brain maturation (see also Ellis et al., 2012, Telzer, 2015). A study by Cohen et al. (2010) illustrates the adaptive character of the adolescent brain. In this study, adolescent participants (ages 14–19) showed a stronger dopaminergic brain response to reward prediction errors when engaging in a learning task than either younger children (ages 8–12) or adults (ages 25–30) (see also Galván et al., 2006, discussed above). Prediction error is considered important in motivating learning (Pagnoni et al., 2002, Schultz et al., 1997) and suggests that adolescents can take advantage of such error feedback as they explore the environment. Although the authors interpreted the finding as consistent with the hypothesis that adolescents engage in riskier behavior than younger or older persons, the task did not involve risky decision-making and thus was also consistent with the conclusion that adolescents are disproportionately primed to take advantage of positive feedback in a learning situation (see also Davidow et al., 2016, Satterthwaite et al., 2012).

Murty et al. (2016) recently proposed a model of experience-based brain development termed the Experience-Driven Adaptive Cognitive Model of adolescence that highlights the role of dopamine activation during adolescence as a modulator of enhanced memory-circuit integration between the hippocampus (HPC) and PFC. They review evidence indicating the importance of this process for building long-term memory representations that enable the use of experience to further more adaptive decision making. In particular, abundant evidence from studies in animals suggests that dopamine release from neurons in the midbrain plays an important role in the coding of reward prediction errors and uncertainty (Fiorillo et al., 2003, Tobler et al., 2005). In humans, such signals play an important role in episodic memory formation (Shohamy and Adcock, 2010), and tonic levels of midbrain dopamine activation may encourage exploration and acquisition of long-term memories that support learning and adaptation (Düzel et al., 2010). As Murty et al. (2016) say, “…different lines of research provide compelling support for adolescence being a unique period of plasticity and refinement of HPC-PFC circuits for the establishment of contextually-relevant responses to guide and optimize goal-oriented behaviors.” (p. 54). Their model is consistent with the suggestion that the exploratory behavior motivated by dopaminergic activation during adolescence serves adaptive purposes.

A study of adolescent decision-making in the presence of reward reversal also supports the adaptive character of the adolescent brain. When confronted with changing reward contingencies, adolescents exhibited heightened activation of insular cortex, which was associated with more rapid reversal learning (Hauser et al., 2015). Young adults were slower to respond to the changes in contingencies. Recognizing such changes in contingencies is evidence of engaged executive function. These results suggest a possible mitigating factor (that adolescent brains are quick to recognize changes in reward contingencies), offsetting to some degree their heightened sensation seeking or attraction to novel experience.

Interestingly, youth with higher sensation seeking exhibit less impulsive choice as they age. In a national sample of adolescents and young adults, Romer et al. (2010) found that high sensation seekers, who were more likely to engage in risky behavior than low sensation seekers, also exhibited higher levels of the ability to delay gratification as they aged, an important indicator of reduced impulsivity and cognitive control (Casey et al., 2011). Indeed, they reached higher levels of patience than youth who were lower in sensation seeking. Thus, experience gained during risk taking can lead to more adaptive decision-making over the long term, especially among those with sufficient cognitive skills, such as WM, to integrate their experience for future behavior.

Risk taking during adolescence has been described as normative. For example, Baumrind (1987) argued that “risk taking behavior characterizes normal adolescent development.” (p. 98) Furthermore, “…some experimentation – be it with drugs or sex or odd diets or new ideas – is typical, and may even be an essential component, of a healthful adolescent experience and contribute to optimal competence.” (p. 98) Some studies have shown that adolescents who experimented with drugs were more socially accepted by peers (Lightfoot, 1997, Maggs et al., 1995) and exhibited better adjustment than those who completely abstained from drug use (Shedler and Block, 1990). Chassin et al. (1989) observed that youth higher in sensation seeking engaged in what they called “constructive” risk taking, characterized by desire for independence and academic success, whereas “destructive” risk takers were characterized by impulsivity and antisocial tendencies. In a longitudinal study across grades 5–10, Lewis-Bizan et al. (2010) observed that youth who were characterized as possessing positive developmental attributes, such as competent control over behavior, were also likely to engage in risky behavior. However, their risk taking did not continue at high levels later in adolescence.

In some statements of imbalance models (e.g., Casey and Caudle, 2013, Spear, 2013), the importance of individual differences in adolescent risk taking is acknowledged. Nevertheless, the lower ability of the adolescent to control socioemotional decisions continues to be cited as a common deficit in adolescent brain function. For example, studies using driving simulation tasks by Steinberg and colleagues (e.g., Chein et al., 2011) are interpreted to show that adolescents’ brains respond impulsively to the presence of their peers (Steinberg, 2014), whereas adults are less susceptible to these influences. Although peer effects may be stronger in adolescents, the direction of such effects appears to depend on the characteristics of those peers. Simply placing adolescents behind the wheel with peers in the vehicle does not necessarily produce riskier driving (see Romer et al., 2014, for a review). In particular, greater risk taking in the presence of peers is consistent with a group polarization effect of peer influence, such that when drivers think peer passengers expect them to drive aggressively, they are more likely to do so. However, when peers are not expected to hold these preferences, adolescent drivers are no more likely to drive in a risky manner (Bingham et al., 2016, Simons-Morton et al., 2014).

It is likely therefore that youth with relatively good executive control and peer groups with similar characteristics will be able to experiment with risky behavior without advancing to more serious outcomes (Lightfoot, 1997). However, as our analysis suggests, some youth will experience premature pregnancy, substance use, and other maladaptive behaviors that adversely affect educational attainment, health, and other social outcomes (Institute of Medicine, 2011).

## 3 Beyond imbalance during adolescence

Despite the valuable insights spurred by imbalance models, it time to move beyond these models to consider the role that experience plays in healthy adolescent development. One potentially fruitful direction in future research would be to compare measures of gist learning and decision making to measures that capture the development of wisdom (Sunstein, 2008; see also, Reyna, 2008, Reyna and Huettel, 2014). Such a direct comparison would test Reyna and Brainerd’s (2011) fuzzy-trace theory, which predicts that decision-making shifts from relying on lower-level (verbatim) representations that encourage risk taking to more abstract (gist) representations that support healthier decisions to categorically avoid catastrophic risks (but to take risks when they offer the possibility of a categorically superior outcome relative to less risky options). In this regard, the theory has already successfully predicted self-reported real-world risky behaviors using gist measures (e.g., Broniatowski et al., 2015, Fraenkel et al., 2012, Mills et al., 2008, Reyna et al., 2011, Reyna and Mills, 2014, Wolfe et al., 2015).

Another promising direction for future research is to examine the relation between executive functions such as WM and the decline in maladaptive risk taking with age. As the consequences of exploratory risk taking accumulate in experience, those with stronger WM should be able to incorporate those experiences more effectively in decisions entailing maladaptive risk. Preliminary evidence for this prediction has been observed in a study of late adolescent risk for drug addiction. Those with stronger WM ability were more able to avoid advancing to drug dependence apart from differences in impulsive tendencies (Khurana et al., 2017).

Our model also suggests that we look at risk taking more broadly than just examining behaviors with adverse consequences. For example, Romer et al. (2016) showed that both sensation seeking and parts of the BAS were related to risk behaviors that are considered adaptive, such as entering scholastic competitions and engaging in sports (see also Hansen and Breivik, 2001). Many of the risky behaviors that adolescents pursue involve potential social conflicts with parents or peers (Weber et al., 2002), and these and other forms of risk behavior are also likely to increase during adolescence and should be considered in our models.

We have said little about sex differences, but as is evident in Fig. 2, there are gender differences in sensation seeking (Cross et al., 2011), which will have implications for different types of risk taking during adolescence. The correlation between sensation seeking and impulsive action is consistent with a smaller but established sex difference in measures of impulsive action (Cross et al., 2011), corresponding to the risk insensitive trajectory in our model in Fig. 5. This trajectory helps to explain the well-established over-representation of males in externalizing behavior, a pattern that begins early in development among youth with weak cognitive control (Bjork and Pardini, 2015, McGue and Iacono, 2005, Moffitt et al., 2011). On the other hand, the small relation between sensation seeking and decisions under known risk is consistent with the lack of sex differences in decisions under known risk (Cross et al., 2011). Nevertheless, the differences in sensation seeking would suggest that females are less inclined to engage in exploratory risk taking. However, many of the rewarding aspects of such behaviors are likely to be domain specific, such that young women may engage in exploration if they perceive the rewards to be sufficiently strong (Romer and Hennessy, 2007, Santos et al., 2016), for example in social domains (Weber et al., 2002). Future research should examine this possibility as well.

Finally, much remains to be learned about the organization of RSNs during the transition to adulthood. It is already known that youth with ADHD have weaker ability to suppress the default mode network (DMN) than normally developing youth (Kessler et al., 2016, Posner et al., 2014). This is evident in stronger connectivity between the DMN and task-positive networks in youth with ADHD. Youth with externalizing disorder and elevated levels of impulsive action exhibit the same pattern (Inuggi et al., 2014; Kessler et al., 2014; Shannon et al., 2011). Future research could identify the neural basis of this deficit and explore potential interventions that could reduce it (Kelly and Castellanos, 2014, Stevens, 2016). These leads could be followed to determine the neural basis of harmful forms of impulsivity as opposed to exploratory forms of risk taking that emerge during adolescence. Research regarding the functional roles of RSNs as they respond to growth in experience and wisdom during the adolescent period would appear to be a fruitful avenue of future research.

As more is learned about the growth of wisdom over the lifespan, it is also important not to overplay the wisdom of adulthood. Just as stereotypes regarding adolescence have colored our interpretation of brain research, it is just as easy to romanticize the experience and wisdom of adulthood. Research shows that relying on gist can lead to predictable biases even in experts (see Wilhelms et al., 2015). The increasing aversion to risk in ambiguous contexts may also lead to less than optimal search tendencies (Tymula et al., 2012). A good deal of research in decision making over the past several decades reveals how heuristics and biases common in adults can produce fallacies in judgment (Kahneman, 2013, Stanovich, 2011). This classic research serves as the foundation of more recent approaches, such as fuzzy-trace theory, that account for fallacies in adulthood but also explain the strengths of mature decision making (Defoe et al., 2014, Reyna et al., 2014).

In conclusion, we have presented an alternative model of adolescent brain development that emphasizes the accumulation of experience as adolescents age and transition to adulthood, with concomitant changes in judgment and decision making (see Table 1 for a summary of differences between the Life-span Wisdom Model and Imbalance Models). The model explains much of the apparent increase in adolescent risk taking as an adaptive need to gain the experience required to assume adult roles and behaviors. The risk-taking that reflects lack of control or excessive sensitivity to immediate rewards is primarily an individual difference that characterizes some persons from an early age that can persist well into adulthood. At the same time, the adolescent brain is supremely sensitive to the learning that can occur during this period and has cognitive capacities to take advantage of the experience gained. The result is a brain with integrated circuits encompassing executive function (i.e., cognitive control and inhibition), as well as verbatim and gist memory networks, which can be called upon to negotiate both novel and familiar situations. The preservation of robust gist thinking maintains wise decision making during later adulthood when cognitive control capacities diminish. We believe this approach is more aligned with the scientific evidence, including results that challenge stereotypes about the adolescent brain.

**Table 1:** Differences between Imbalance Models and Lifespan Wisdom Model.

Imbalance Model	Life-span Wisdom Model
Slower development of PFC and its connection with limbic system results in imbalance that outweighs cognitive control over impulsive urges during adolescence (Fig. 1).	Cognitive control and dopaminergic activation rise in tandem during adolescence; much of adolescent risk taking is exploratory in keeping with the role of dopamine as a signal for novel reward (Fig. 6).
Rise in risk taking and incidence of health compromising behavior during adolescence reflects developmental imbalance.	Risk taking takes at least three forms, with different developmental trajectories (Fig. 5). The form most closely associated with imbalance reflects insensitivity to risk and applies primarily to youth with early elevated levels of impulsive behavior.
Peak in sensation seeking during adolescence produces more risk taking than in children or adults.	Peak in sensation seeking during adolescence motivates greater exploration in ambiguous environments, but risk taking declines monotonically from childhood to adulthood when risks are known, per greater reliance on gist and increasing executive function (Fig. 5).
Imbalance leads to increased injury and maladaptive outcomes during adolescence.	Timing of many maladaptive outcomes occurs in early adulthood when imbalance should be minimal; maladaptive outcomes are more related to high levels of impulsivity combined with risk opportunity and inexperience than to developmental imbalance.
Socioemotional influences excite the dopaminergic system and promote risk taking.	Socioemotional influences can promote risk taking, but social experience (interacting with peers) and positive social influences can promote healthy risk avoidance.
Main emphasis on brain maturation, rather than experience or interventions that can promote adaptive brain development. No predictions about life-span cognitive control or increase in wisdom.	Acknowledges brain maturation that reflects growth in experience and potential interventions to promote healthy decision making by increasing reliance on experience and wisdom.

## Conflict of Interest

None.

## Footnotes

1 In a recent review of imbalance research, Casey (2015) prefers not to describe imbalance as a “deficit” but rather a “brain that is sculpted by evolutionarily based biological constraints….” Nevertheless, these constraints are seen as contributing to a “200% increase in preventable deaths (accidents, suicide, homicide)…” during adolescence (p. 296–297).
